# iFGF23 Plasma Levels in Transfusion-Dependent β-Thalassemia: Insights into Bone and Iron Metabolism

**DOI:** 10.3390/jcm14061834

**Published:** 2025-03-08

**Authors:** Alberto Gobbo, Filomena Longo, Camilla Alice Cattaneo, Martina Verrienti, Gianluca Marzi, Fatima Chamekh, Martina Culcasi, Alberto Cossu, Maria Chiara Zatelli, Maria Rosaria Ambrosio

**Affiliations:** 1 Section of Endocrinology, Geriatrics and Internal Medicine, Department of Medical Sciences, University of Ferrara, 44121 Ferrara, Italy; alberto.gobbo@edu.unife.it (A.G.); ztlmch@unife.it (M.C.Z.); 2 Department of Specialized Medicine, Day Hospital of Thalassemia and Hemoglobinopathies, Azienda Ospedaliero Universitaria S. Anna, 44124 Ferrara, Italy; 3 Unit of Endocrinology and Metabolic Diseases, Department of Specialty Medicines, Azienda Ospedaliero Universitaria di Ferrara, 44124 Ferrara, Italy; martina.verrienti@ospfe.it; 4 Radiology Unit, Azienda Ospedaliero Universitaria di Ferrara, 44124 Ferrara, Italy

**Keywords:** beta-thalassemia, transfusion dependent, hemoglobinopathy, iron load, FGF23, phosphate, calcium, ferritin, iron chelation

## Abstract

**Background:** FGF23 is a phosphate homeostasis regulator; the literature suggests a link between FGF23, iron homeostasis and erythropoiesis. Little is known about the FGF23 level variations in β-thalassemia (βT), which is characterized by ineffective erythropoiesis and iron overload. Our cross-sectional study aims to evaluate the iFGF23 level variations in a large cohort of βT patients considering their bone mineral densities (BMDs) and iron loads. **Methods**: Clinical, biochemical and radiological data were collected from 213 transfusion-dependent βT (TDT) adults referring to the Regional HUB Centre for Thalassaemia and Haemoglobinopathies in Ferrara, Italy. The iFGF23 levels in the TDT patients were compared to the general population’s reference range. The BMDs and hearth and liver iron deposits were assessed with DEXA scans and MRI, respectively. **Results**: The iFGF23 distribution in the TDT subjects is significantly different from that of the general population. The iFGF23 levels are positively correlated with the age at transfusion initiation and calcium and phosphate levels and are negatively correlated with the osteocalcin plasma levels. Patients treated with deferasirox had lower iFGF23 levels than those treated with other chelators. The iFGF23 levels are not correlated with the BMD or iron status. **Conclusions**: These findings provide insights into the relationship between the iFGF23 and bone and iron metabolism in TDT patients. Further studies are needed to explore its potential clinical relevance.

## 1. Introduction

Thalassemic disorders are genetic defects causing the reduced or absent synthesis of one or more hemoglobin chains [1]. Beta-thalassemia (βT), caused by mutations in the β globin genes, displays variable clinical severity depending on the mutation type and residual β globin chain production, divided into transfusion-dependent (TDTs) and non-transfusion-dependent (NTDTs) phenotypes [2]. βT is characterized by ineffective erythropoiesis, which stimulates intestinal iron absorption leading to iron overload and iron-induced oxidative stress over time. In addition, the reduction in erythroid progenitors and the erythrocytic lifespan may require frequent blood transfusions with consequent increases in the tissue iron load [2]. Iron-induced oxidative stress, chronic anemia, bone marrow expansion and extramedullary erythropoiesis play important roles in βT-associated complications and multi-organ function impairment. Besides regular transfusions, conventional βT treatment includes iron chelation therapies, hydroxyurea administration, splenectomy and hematopoietic stem cell transplantation [3].

Endocrine glands are extremely sensitive to iron-induced oxidative stress. Indeed, endocrine dysfunctions are the most frequent complications in adequately transfused and chelated βT individuals [4,5,6,7]. These conditions might be reversible after chelation therapy optimization and iron load normalization, especially when promptly diagnosed [8].

Reduced bone mineral density (BMD) is a common finding in βT, with a prevalence of up to 50% in well-treated βT individuals and even higher in older patients or those receiving inadequate treatment [7,9]. The fracture risk in βT individuals is elevated compared to the general population, and multiple factors contribute to the development of thalassemic bone disease (TBD). Specifically, bone marrow expansion, hemosiderosis, chelation therapy toxicity, hypercalciuria, reduced physical activity and genetic predisposition have all been linked to TBD [9,10]. Additionally, several βT-related endocrinopathies negatively affect bone health, including hypogonadism, diabetes mellitus, GH deficiency, hypoparathyroidism and hypothyroidism [9,10,11]. In recent years, the pharmacological landscape of osteoporosis treatment has expanded, enabling clinicians to manage severe cases with potent anti-osteoporotic drugs. However, the efficacy and safety of these newer treatments have not been extensively investigated in βT individuals, and TBD still remains a challenging condition [9,12].

FGF23 is a potent phosphate and calcium homeostasis regulator, as underlined by the evidence that FGF23 gene mutations cause autosomal-dominant hypophosphatemic rickets (ADHR) [13]. Biologically active, full-length and intact FGF23 (iFGF23) is cleaved by Furin into inactive N- and C-terminal fragments [14,15]. Osteoblasts and osteocytes secrete iFGF23, which targets several organs interacting with four tyrosine kinase receptors [14,16,17]. In the kidneys, parathyroid and brain, FGF23 canonical signaling occurs in the presence of the co-receptor alpha-Klotho (KL) [18,19]. KL-dependent pathways are responsible for phosphate urinary excretion, calcium urinary reabsorption, a reduction in PTH secretion and the suppression of active 1,25(OH)_2_ vitamin D synthesis by the kidneys [14,15,20,21,22,23,24]. Despite the fact that the direct FGF23 effects on bone turnover are not well characterized, FGF23 has been shown to inhibit tissue-non-specific alkaline phosphatase through a KL-independent mechanism, possibly contributing to mineralization defects [25,26]. FGF23 also negatively regulates erythropoiesis and erythropoietin (Epo) secretion [27], which, in turn, enhances FGF23 transcription and cleavage, resulting in increased C-terminal FGF23 (cFGF23) levels [28,29,30]. The interplay between iron status and FGF23 production and degradation is highly complex and finely regulated, resulting in phosphate homeostasis maintenance [16].

In iron-deficient individuals, the cFGF23 levels increase while the iFGF23 levels are stable, suggesting that FGF23 cleavage is directly stimulated in this condition [31,32,33]. Additionally, in the presence of cleavage preventing FGF23 gene mutations, as seen in ADHR, iron deficiency is associated with an increase in the iFGF23 levels, which consequently affect the serum phosphate levels [32,33]. This suggests that iron deficiency directly stimulates both FGF23 expression and cleavage. Interestingly, specific intravenous iron formulations have been shown to reduce the cFGF23 levels and transiently increase the iFGF23 levels in iron-deficient patients, leading to temporary hypophosphatemia [31,34,35,36,37]. Hepcidin is an acute-phase protein which plays a key role in the development of functional iron deficiency during inflammation by reducing intestinal iron absorption and iron release from body stores [38]. Since iron deficiency stimulates both FGF23 expression and cleavage, hepcidin-mediated functional iron insufficiency influences the FGF23 levels in a similar fashion [39]. However, hepcidin does not appear to directly affect FGF23 metabolism, as demonstrated in murine models by Hanudel et al. [40]. Conversely, Higashimoto et al. demonstrated that in vitro hepcidin expression is modulated by FGF23 in a dose-dependent manner [41].

Since FGF23 is involved in iron metabolism and erythropoiesis, its levels might be influenced by iron overload and ineffective erythropoiesis, as observed in βT. Indeed, Tangngam et al. found that the iFGF23 levels were significantly lower in TDT children and adolescents as compared to those of healthy controls, suggesting that iron accumulation may impair FGF23 production [42]. On the contrary, Stefanopoulos et al. reported similar iFGF23 levels in βT adults and healthy controls [43]. Furthermore, Aprile et al. observed significantly higher total FGF23 levels but no differences in the iFGF23 levels in a cohort of 40 βT patients compared to healthy hematopoietic stem cell donors [30]. These discrepancies in the iFGF23 levels among βT subjects may be attributed to variations in the assay methodologies and differences in the study population characteristics.

Our cross-sectional study aims at evaluating the iFGF23 plasma level variations in a large cohort of βT patients according to their bone mineral densities (BMDs) and iron loads.

## 2. Materials and Methods

We collected data from 213 transfusion-dependent βT adult patients, excluding patients younger than 18 years old or with severe renal dysfunction (eGFR < 30 mL/min) and those who underwent bone marrow transplantation, referring from 1st January to 31st December 2023 to the Regional HUB Centre for Thalassaemia and Haemoglobinopathies of Ferrara, Italy. All patients received regular blood transfusions, maintaining the pre-transfusion hemoglobin (Hb) > 9.5 g/dL, according to international guidelines and national Good Clinical Practices from the Italian Society of Thalassemia and Hemoglobinopathies (SITE) [44,45].

### 2.1. Clinical and Biochemical Data

We recorded the age, sex, chelation regimen and type of chelators [deferasirox (DFX), deferiprone (DFP), deferoxamine (DFO)], age at transfusion initiation (in months), weight, height, BMI and history of splenectomy.

We recorded the blood levels of creatinine, urea, calcium, phosphate, parathormone (PTH), 25-OH vitamin D, magnesium, zinc, alkaline phosphatase, bone turnover markers [bone alkaline phosphatase (BAP), C-terminal telopeptide (Ctx), and osteocalcin], insulin-like growth factor 1 (IGF1), thyroid-stimulating hormone (TSH), serum iron, transferrin, soluble transferrin receptor (sTfR), ferritin and Epo. Urinary levels of phosphate, calcium, creatinine and proteins were assessed on a 24 h urinary sample. The glomerular filtration rate (GFR) was estimated with the Cockcroft–Gault equation.

These assessments are included in the βT annual evaluation according to the SITE Good Clinical Practices [6]. Blood and urine samples were obtained before a scheduled blood transfusion.

### 2.2. FGF23 Assay

We measured the EDTA plasma iFGF23 levels by the chemiluminescence immunoassay “LIAISON^®^ FGF 23 test” obtained from DiaSorin (Saluggia, Italy), which has a Limit of Detection of 5 pg/mL and a Limit of Quantification of 6.5 pg/mL. The kit provides normal reference values of iFGF23 in a European control population of 910 adult individuals 18–89 years old and with an eGFR > 60 mL/min (median iFGF23: 57.5 pg/mL; 2.5–97.5° percentile interval = 23.2–95.4 pg/mL).

### 2.3. Radiological Data

The BMD of the lumbar spine, hip and femoral neck (LS-BMD, F-BMD and FN-BMD, respectively) were assessed with a HOLOGIC^®^ DXA scan (Hologic, Inc., Marlborough, MA, USA) performed ± 18 months from the iFGF23 measurement.

Tissue iron deposits were assessed by MRI using a 1.5 T scanner (Siemens Healthineers, Erlangen, Germany), recording the heart and liver T2*. The Liver Iron Concentration (LIC) was derived from liver T2* measurement.

## 3. Statistical Analysis

A binomial test was used to assess whether the iFGF23 levels in the TDT patients were included within the 2.5th and 97.5th percentiles of the iFGF23 levels in the reference general population. The Shapiro–Wilk test was used to test the normality of the continuous variables. Comparisons of the non-parametric continuous variables between two groups was performed with the Mann–Whitney U test. The Kruskal–Wallis test was employed to compare the non-parametric continuous variables between multiple groups, and post hoc analyses were performed with Dwass–Steel–Critchlow–Fligner pairwise comparisons. The Benjamini–Hochberg correction was applied for comparisons of more than two groups. Non-parametric continuous variables are expressed as median values and interquartile ranges (IQRs). Univariable and multivariable linear regression models were fitted to evaluate the relationship between the independent variables and iFGF23 levels. A *p*-value < 0.05 is considered statistically significant. We performed the analyses using Excel software and Jamovi software (The jamovi project, 2023; version 2.4) [46]. Graphics were created with R-Studio (R-studio version 2024.4.1.748, R version 4.2.2).

## 4. Results

Among the 213 enrolled TDT patients, 117 individuals were females and 96 were males. Table 1 and Table 2 show clinical, laboratory and radiological data of the study population.

**Figure 1:**
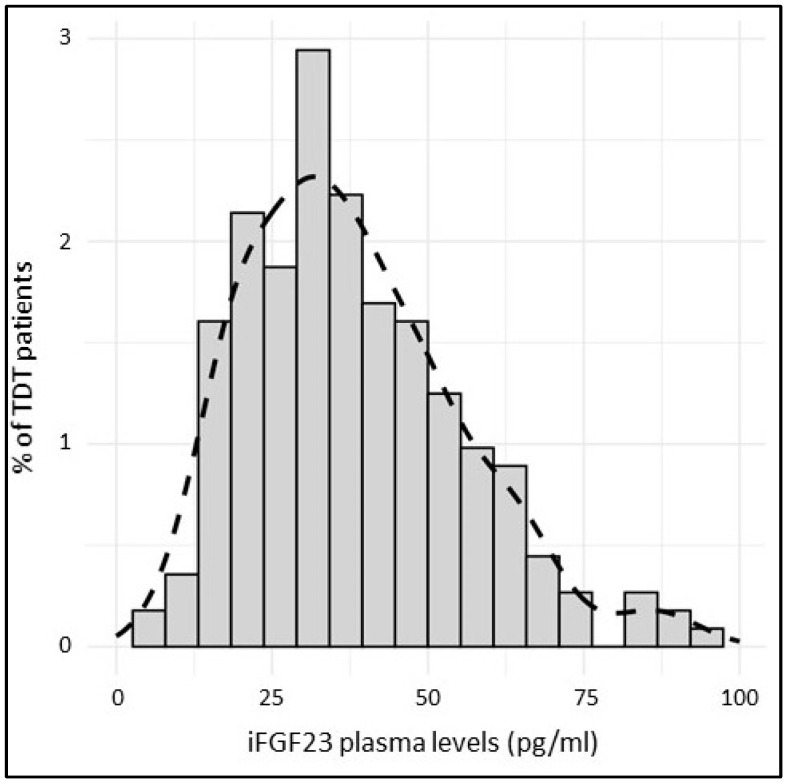
iFGF23 levels in TDT population.

The overall iFGF23 distribution did not follow a normal distribution but presented a right-sided tail, as confirmed by the Shapiro–Wilk test (*p* < 0.001, Figure 1).

In our cohort, 78% of the iFGF23 values were included within the 2.5th and 97.5th percentiles of the general population range (i.e., 23.2–95.4 pg/mL), indicating that the iFGF23 distribution in the TDT subjects was significantly different compared to that of the general population (*p* < 0.001). The latter showed higher iFGF23 plasma levels compared to those of the TDT patients.

The univariate linear regression models showed that the iFGF23 levels were significantly positively associated with age, age at transfusion initiation, BMI, history of splenectomy, and plasma levels of calcium, phosphate and sTfR. On the contrary, they were negatively correlated with the magnesium, osteocalcin, BAP and IGF1 plasma levels and 24 h urinary protein and calcium levels. BMDs and iron deposits were not associated with the iFGF23 levels (Table 3).

As shown in Table 4, the multivariate linear regression model confirmed that the iFGF23 levels are significantly positively correlated with the age at transfusion initiation, calcium and phosphate, while they are negatively correlated with the osteocalcin plasma levels. These associations are independent of age, BMI and renal function.

**Figure 2:**
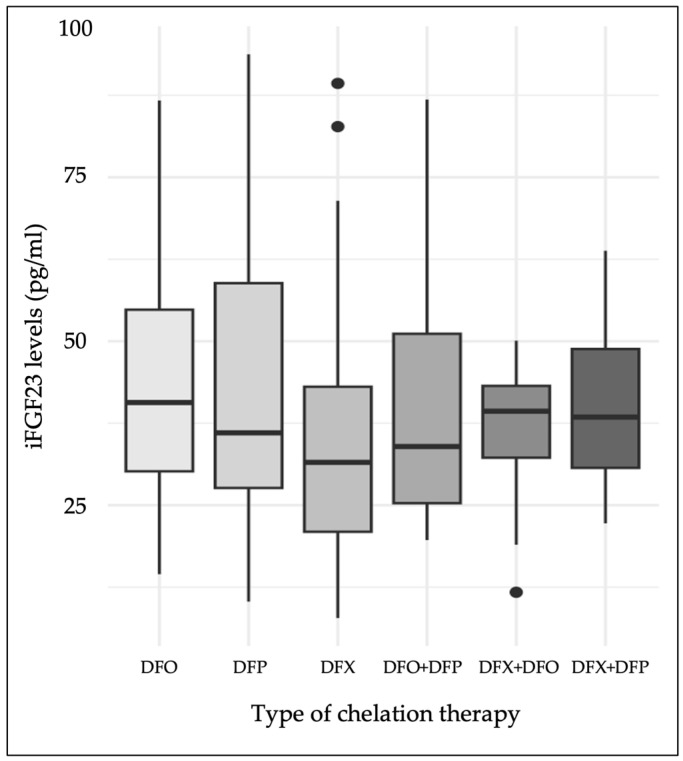
iFGF23 levels according to chelation therapy. DFO: deferoxamine; DFP: deferiprone; DFX: deferasirox. Median iFGF23 levels are shown in pg/mL with IQRs according to treatment group.

We divided the TDT patients into six groups according to the type of chelation therapy (see Table 1). The iFGF23 levels differed significantly among the treatment groups (χ^2^ = 11.35, df = 5, *p* = 0.045). The DFX treatment group presented lower iFGF23 levels compared to the other treatments; however, none of the pairwise comparisons were significant after *p*-value adjustment (Figure 2). After grouping all the patients according to DFX therapy vs. other chelation treatments, the former group presented significantly lower iFGF23 levels (median iFGF23 levels: 32.1 [22.4–43.3] vs. 38.8 [28.4–55.7] pg/mL, respectively, *p =* 0.004) and significantly higher phosphate levels (median phosphate levels: 3.8 [3.4–4.2] vs. 3.5 [3.2–3.9] mg/dL, respectively, *p =* 0.01). Individuals on DFX showed similar eGFR and 24 h urinary proteins and phosphate levels compared to those on other chelation treatments.

## 5. Discussion

Our study shows that the iFGF23 plasma levels in a large cohort of TDT patients are not associated with their BMDs or iron loads, but they vary according to the type of chelation treatment, age at transfusion initiation as well as osteometabolic biochemical parameters such as the phosphate, calcium and osteocalcin plasma levels. In addition, we found that the iFGF23 levels in the TDT subjects were lower than those detected in the reference population. These results provide novel insights into the osteometabolic network linked to FGF23.

TBD is one of the most frequent and challenging βT endocrine complications, driven by multiple pathophysiologic mechanisms that often lead to severe clinical manifestations [9,10]. While the literature provides evidence on the link between FGF23, iron homeostasis and erythropoiesis, little is known about the FGF23 level variations in patients with ineffective erythropoiesis and iron overload, such as those with βT [16].

We observed that the iFGF23 levels in the TDT patients were significantly lower as compared to those of the reference population, suggesting that FGF23 regulation is altered in TDT patients as compared to healthy individuals.

Our findings are consistent with those of Tangngam et al., who reported significantly lower iFGF23 levels in TDT children and adolescents compared to those of healthy controls, suggesting that iron accumulation in bones may impair FGF23 production [42]. In contrast, Aprile et al. and Stefanopoulos et al. found similar iFGF23 levels in βT adults compared to healthy controls [30,43]. These discrepancies may be attributed to differences in the assay methods and study population characteristics. Indeed, previous studies evaluated the iFGF23 levels in different matrices (plasma vs. serum) with ELISAs from different manufacturers. There is evidence that iFGF23 is less stable in serum compared to plasma, leading to time-dependent decreases in its levels [47]. These methodological differences may contribute to the variability observed across studies. Additionally, differences in patient selection may have contributed to this variability. For instance, the cohort studied by Aprile et al. included a mixed βT population, with both TDT and NTDT patients, as well as individuals not receiving chelation therapy. Since NTDT patients may exhibit iFGF23 levels comparable to those of the general population, especially in milder disease forms, this heterogeneity could have influenced their results.

FGF23 plays a key role in maintaining the phosphate balance in response to chronic changes in the serum phosphate levels [48]. As expected, our data confirm a positive association between phosphate and iFGF23 levels, indicating phosphate as a major regulator of FGF23 production and secretion in TDT patients, similar to healthy subjects. Additionally, we found a positive association between the calcium and iFGF23 levels, supporting the hypothesis that calcium may stimulate FGF23 secretion [49].

The osteocalcin levels, a marker of bone turnover [50], were negatively associated with the iFGF23 levels in our cohort. This suggests a link between iFGF23 levels and bone metabolism. However, in our TDT cohort, there was no correlation between the iFGF23 levels and FN- and LS-BMD, in contrast to previous reports showing a negative association between the iFGF23 levels and LS-BMD assessed by DEXA in βT-major patients [51]. Confounding factors such as comorbidities and osteoporosis treatment may have influenced our findings, warranting cautious interpretation. Previous evidence showed that the iFGF23 levels are negatively correlated with the BMD in end-stage renal disease patients [52], suggesting that FGF23 may predict bone loss. Our data do not support this hypothesis in TDT patients, wherein the FGF23-related osteometabolic network is likely disrupted. In addition, DEXA has shown limitations in the evaluation of bone health in βT-major patients due to bone architecture characteristics and deformities, especially at the lumbar spine, causing assessment errors [53,54].

Previous studies suggest that FGF23 synthesis is regulated by PTH, although the precise mechanism remains unclear [49]. Ito et al. demonstrated that PTH directly stimulates FGF23 expression in murine osteocyte-like cells in vitro [55]. On the contrary, low PTH levels indirectly increase iFGF23 levels by causing hyperphosphatemia [56]. Interestingly, hypoparathyroid βT patients display lower iFGF23 levels compared to those of βT patients with normal parathyroid functioning [42]. Severe vitamin D deficiency has also been reported to suppress iFGF23 production [48]. However, in our population, the iFGF23 levels were not associated with the vitamin D nor PTH levels, suggesting a disruption in the interplay between these hormones in TDT. Further studies evaluating the 1,25(OH)_2_ vitamin D levels in TDT patients will clarify this issue. Moreover, the PTH and vitamin D influence on FGF23 levels could be attenuated by TDT-associated issues, such as the chelation treatment or age of transfusion initiation.

Saki et al. proposed that iFGF23 secretion may be directly stimulated by ferritin [57]. In contrast, we did not find any association between the iFGF23 and ferritin levels, likely due to an optimal control of the iron burden as witnessed by the lower ferritin levels in our population as compared to those found by Saki et al.

As mentioned before, we observed a positive association between the iFGF23 levels and age at transfusion initiation, suggesting that the disease severity may influence FGF23 levels. Indeed, Tangngam et al. suggested that bone iron deposits may impair osteocyte functioning and FGF23 secretion [42]. However, our data failed to show any association between iFGF23 and iron deposits evaluated by MRI. Additionally, the Epo levels were not associated with the iFGF23 levels, in contrast with Aprile et al., who found that Epo stimulates FGF23 secretion in βT [30].

In our population, patients treated with DFX exhibited lower iFGF23 levels compared to those on other chelators, particularly DFO. DFX is known to induce renal proximal tubular dysfunction [58,59,60], which, in turn, is a recognized cause of hypophosphatemia and reduced iFGF23 levels [48]. We also found a negative association between the iFGF23 levels and 24 h urinary proteins, which was not confirmed by multivariate analyses. DFX therapy was not associated with renal impairment nor tubulopathy evaluated by the eGFR and 24 h urinary protein and phosphate levels. Although renal impairment and tubulopathy influence iFGF23 levels, our data do not confirm this association, and the limited sample size prevents us from drawing stronger conclusions on the DFX role. Moreover, a direct effect of DFX on osteocyte functioning, including iFGF23 production and secretion, cannot be excluded.

A strength of this study is the population homogeneity. Indeed, all individuals followed a regular transfusion regimen to maintain pre-transfusion hemoglobin levels > 9.5 g/dL, according to TIF guidelines and SITE Good Clinical Practices [44,45]. In addition, all patients were well chelated and followed with a multidisciplinary approach at a single Italian Centre. Therefore, our results can be applied only to populations with similar characteristics, representing a possible pitfall of our conclusions. A further limitation of this study is the absence of a control group for direct comparison with TDT patients. Nevertheless, we were able to rely on the reference values of the iFGF23 assay kit, which were validated in a population of healthy European individuals. Future studies should evaluate additional parameters, such as the use of bone-targeting agents, renal tubular phosphate reabsorption, plasma levels of soluble Klotho, C-terminal FGF23, 1,25(OH)_2_ vitamin D, sclerostin and hepcidin to enhance our understanding of the FGF23 physiology in TDT patients. Moreover, a prospective study might be useful to further explore the impact of chelation treatment with DFX on iFGF23 levels.

## 6. Conclusions

In conclusion, this study highlights that the iFGF23 levels in TDT patients are influenced by the disease severity and chelation treatment. Phosphate remains a key FGF23 regulator in this clinical setting, while calcium and osteocalcin might also play roles. Although no direct association between the iFGF23 levels and BMD was observed in our cohort, the findings suggest a potential link between FGF23 and bone metabolism. However, our data do not support iFGF23 levels as a bone loss marker in TDT.

Establishing a TDT-specific reference range for FGF23 would aid in interpreting biochemical data and improving the understanding of the FGF23 role in thalassemia-associated endocrine complications, particularly thalassemic bone disease.

Our study has both scientific and clinical implications. Indeed, future research on FGF23 in TDT should consider the potential regulatory factors we identified in this study. Moreover, our findings can help physicians in interpreting biochemical data in a clinical setting.

## Figures and Tables

**Table 1:** TDT population’s clinical data. DFO: deferoxamine; DFP: deferiprone; DFX: deferasirox.

Clinical Data		
	N	%
Sex:		
Females	117	54.9%
Males	96	45.1%
Splenectomy:		
Yes	136	63.8%
No	77	36.2%
Chelation therapy:		
DFX	87	40.8%
DFO	46	21.6%
DFP	39	18.3%
DFX + DFO	13	6.1%
DFX + DFP	8	3.8%
DFO + DFP	20	9.4%

**Table 2:** TDT population’s laboratory and radiological data. BAP: bone alkaline phosphatase; Ctx: beta-crosslaps; Epo: erythropoietin; IGF1: insulin-like growth factor 1; sTfR: soluble transferrin receptor; LS/F/FN-BMD: lumbar spine/total hip/femoral neck bone mass density.

Laboratory and Radiological Data		
	Median	IQR
iFGF23 (pg/mL)	36.0	[24.9, 48.8]
Age (years)	50.0	[44.0, 54.0]
Age at transfusion initiation (months)	12.0	[6.00, 36.0]
BMI (kg/m^2^)	22.4	[20.4, 24.6]
eGFR (mL/min)	91.8	[74.8, 112]
24 h urinary proteins (mg/die)	160	[105, 234]
24 h urinary creatinine (g/die)	1.00	[0.800, 1.30]
24 h urinary calcium (mg/die)	291	[202, 403]
24 h urinary phosphate (g/die)	0.700	[0.500, 0.900]
ALP (U/L)	75.5	[61.0, 96.0]
Urea (mg/dL)	42.0	[35.0, 50.0]
Creatinine (mg/dL)	0.735	[0.610, 0.880]
Calcium (mg/dL)	9.50	[9.10, 9.80]
Phosphate (mg/dL)	3.65	[3.20, 4.10]
Serum iron (μg/dL)	231	[206, 267]
Magnesium (mg/dL)	2.15	[1.99, 2.31]
Epo (mUI/mL)	49.9	[29.1, 73.1]
PTH (pg/mL)	26.0	[19.0, 37.0]
25-OH vitamin D (ng/mL)	29.8	[22.3, 36.1]
BAP (μg/L)	16.9	[12.7, 23.6]
Ctx (ng/mL)	0.237	[0.146, 0.405]
Osteocalcin (ng/mL)	18.7	[15.0, 22.1]
IGF1 (ng/mL)	79.7	[57.0, 99.8]
TSH (μU/mL)	2.16	[1.56, 2.89]
Zinc (μg/mL)	86.0	[77.0, 95.5]
Transferrin (mg/dL)	167	[151, 180]
sTfR (mg/L)	3.25	[2.33, 4.25]
Ferritin (ng/mL)	536	[339, 950]
MRI parameters:		
Heart T2* (ms)	43.0	[39.0, 45.0]
Liver T2* (ms)	8.57	[4.24, 15.8]
LIC	3.21	[1.81, 6.49]
DXA parameters:		
LS BMD	0.800	[0.732, 0.894]
F BMD	0.719	[0.640, 0.818]
FN BMD	0.609	[0.534, 0.684]

**Table 3:** iFGF23 plasma level association with clinical, biochemical and radiological parameters assessed by univariate linear regression. BAP: bone alkaline phosphatase; Ctx: beta-crosslaps; DFO: deferoxamine; DFP: deferiprone; DFX: deferasirox; Epo: erythropoietin; IGF1: insulin-like growth factor 1; sTfR: soluble transferrin receptor; LS/F/FN-BMD: lumbar spine/total hip/femoral neck bone mass density. Significant associations are indicated with a †.

Predictor	Estimate (β)	Standard Error	95% CI	*p*-Value
Sex (males–females)	3.52	2.37	[−1.14, 8.19]	0.138
Age †	0.48	0.13	[0.23, 0.73]	<0.001
Age at transfusion initiation †	0.021	0.007	[0.007, 0.035]	0.004
BMI †	0.94	0.37	[0.22, 1.66]	0.011
Splenectomy †	4.95	2.44	[0.14, 9.76]	0.044
Phosphate †	5.81	1.6	[2.66, 8.97]	<0.001
Calcium †	4.86	2.17	[0.57, 9.14]	0.027
BAP †	−0.24	0.092	[−0.42, −0.062]	0.009
Osteocalcin †	−0.39	0.15	[−0.69, −0.092]	0.011
Ctx	−7.14	4.26	[−15.5, 1.26]	0.095
ALP	−0.067	0.035	[−0.14, 0.002]	0.057
PTH	0.039	0.079	[−0.12, 0.2]	0.627
25-OH vitamin D	0.16	0.11	[−0.065, 0.38]	0.165
Magnesium †	−11.7	3.97	[−19.6, −3.92]	0.003
Zinc	−0.079	0.06	[−0.19, 0.03]	0.164
IGF1 †	−0.094	0.031	[−0.155, −0.033]	0.003
TSH	0.96	0.66	[−0.34, 2.25]	0.147
24 h urinary proteins †	−0.024	0.009	[−0.042, −0.005]	0.01
24 h urinary calcium †	−0.026	0.008	[−0.042, −0.009]	0.003
24 h urinary phosphate	−3.89	2.27	[−8.37, 0.59]	0.089
24 h urinary creatinine	2.91	3.38	[−3.76, 9.58]	0.39
eGFR	0.052	0.039	[−0.025, 0.13]	0.185
Creatinine	7.43	6.34	[−5.06, 19.9]	0.242
Urea	0.177	0.098	[−0.017, 0.371]	0.073
Epo	0.007	0.013	[−0.019, 0.033]	0.594
Transferrin	0.09	0.05	[−0.01, 0.19]	0.09
sTfR †	2.35	0.87	[0.65, 4.06]	0.007
Ferritin	−0.0009	0.002	[−0.004, 0.002]	0.555
Serum iron	−0.046	0.023	[−0.092, 0.0003]	0.052
Chelation therapy:				
DFP-DFX †	7.57	3.27	[1.31, 14.2]	0.022
DFO-DFX †	9.26	3.09	[3.16, 15.4]	0.003
DFO + DFP-DFX	6.52	4.21	[−1.78, 14.8]	0.123
DFX + DFO-DFX	1.87	5.05	[−8.08, 11.8]	0.711
DFX + DFP-DFX	6.59	6.27	[−5.77, 19]	0.294
MRI parameters:				
LIC	−0.073	0.22	[−0.52, 0.37]	0.746
Liver T2*	−0.264	0.16	[−0.59, 0.06]	0.11
Heart T2*	0.039	0.14	[−0.24, 0.32]	0.782
DXA scan				
LS-BMD	16	8.47	[−0.71, 32.7]	0.06
F-BMD	14.4	8.98	[−3.27, 32.2]	0.11
FN-BMD	9.87	10.9	[−11.6, 31.3]	0.37

**Table 4:** iFGF23 plasma level association with clinical, biochemical and radiological parameters assessed by multivariate linear regression. BAP: bone alkaline phosphatase; DFO: deferoxamine; DFP: deferiprone; DFX: deferasirox; IGF1: insulin-like growth factor 1; sTfR: soluble transferrin receptor. Significant associations are indicated with a *.

Predictor	Estimate (β)	Standard Error	95% CI	*p*-Value
Age	0.245	0.224	[−0.199, 0.689]	0.276
Age at transfusion initiation *	0.022	0.009	[0.004, 0.040]	0.015
BMI	0.467	0.451	[−0.424, 1.359]	0.302
Splenectomy	−2.851	3.077	[−8.938, 3.237]	0.356
eGFR	0.004	0.055	[−0.106, 0.114]	0.944
Calcium *	7.283	2.919	[1.508, 13.058]	0.014
Phosphate *	5.402	1.835	[1.771, 9.034]	0.004
Magnesium	−6.875	4.439	[−15.657, 1.908]	0.124
Osteocalcin *	−0.697	0.241	[−1.173, −0.221]	0.004
BAP	0.146	0.138	[−0.127, 0.420]	0.291
sTfR	1.711	1.229	[−0.719, 4.142]	0.166
IGF1	−0.061	0.047	[−0.153, 0.032]	0.196
24 h urinary proteins	−0.017	0.010	[−0.038, 0.004]	0.103
24 h urinary calcium	−0.012	0.009	[−0.030, 0.006]	0.186
Chelation therapy:				
DFP-DFX	5.639	3.759	[−1.800, 13.077]	0.136
DFO-DFX *	7.935	3.953	[0.114, 15.757]	0.047
DFO + DFP-DFX *	13.716	5.172	[3.483, 23.949]	0.009
DFX + DFO-DFX	−0.502	5.902	[−12.181, 11.176]	0.932
DFX + DFP-DFX	4.774	6.314	[−7.720, 17.268]	0.451

## Data Availability

The original contributions presented in this study are included in the article. Further inquiries can be directed to the corresponding author(s).
